# Self-Reported Sleep Disturbance is an Independent Predictor of All-Cause Mortality and Respiratory Disease Mortality in US Adults: A Population-Based Prospective Cohort Study

**DOI:** 10.3389/ijph.2023.1605538

**Published:** 2023-02-14

**Authors:** Xinran Hou, Jiajia Hu, E Wang, Jian Wang, Zongbin Song, Jie Hu, Jian Shi, Chengliang Zhang

**Affiliations:** ^1^ Department of Anesthesiology, Xiangya Hospital, Central South University, Changsha, China; ^2^ National Clinical Research Center for Geriatric Disorders, Xiangya Hospital, Central South University, Changsha, China; ^3^ Department of Anesthesiology, Changsha Yamei Plastic Surgery Hospital, Changsha, China; ^4^ Department of Cardiovascular Surgery, Xiangya Hospital, Central South University, Changsha, China

**Keywords:** sleep disturbance, NHANES, NDI, all-cause mortality, disease-specific mortality

## Abstract

**Objective:** Self-reported sleep disturbance is common but its association with mortality has rarely been investigated.

**Methods:** This prospective cohort analysis included 41,257 participants enrolled in the National Health and Nutrition Examination Survey from 2005 to 2018. Self-reported sleep disturbance in the present study refers to the patients who have ever consulted doctors or other professionals for trouble sleeping. Univariate and multivariate survey-weighted Cox proportional hazards models were used to evaluate the association of self-reported sleep disturbance with all-cause and disease-specific mortality.

**Results:** Approximately 27.0% of US adults were estimated to have self-reported sleep disturbance. After adjusting for all sociodemographic variables, health behavioral factors, and common comorbidities, participants with self-reported sleep disturbance tend to have higher all-cause mortality risk with a hazard ratio (HR) of 1.17 (95% CI, 1.04–1.32) and chronic lower respiratory disease mortality risk (HR, 1.88; 95% CI, 1.26–2.80), but not cardiovascular disease mortality risk (HR, 1.19; 95% CI, 0.96–1.46) and cancer mortality risk (HR, 1.10; 95% CI, 0.90–1.35).

**Conclusion:** Self-reported sleep disturbance could be associated with higher mortality in adults, and may need to be paid more attention in public health management.

## Introduction

Sleep is a fundamental biological process integral to human health, and sleep problems are linked with cognitive, metabolic, cardiovascular, and immunological impairment (1). Sleep duration is the most common indicator to evaluate sleep and numerous epidemical studies have demonstrated that both short and long sleep duration could be a causal risk factor for mortality (2–4) or major cardiovascular events (5–7). However, the measurement bias of sleep duration affects the robustness of its association with mortality risk (8), and the optimal sleep duration varies from person to person, since some individuals may complain of trouble sleeping, sleepiness, or anxiety even if they spend enough time in bed (9, 10). Moreover, to confirm the diagnosis of a certain type of sleep disorder, a series of questionnaires and examinations (e.g., polysomnography) (11, 12) are necessary, which limits the evaluation of sleep health in the general population. On the other hand, subjective sleepiness is valid to indicate the drive for sleep (13) and is more effective in predicting voluntary decreases in social activity than sleep duration (10). Excessive daytime sleepiness is associated with high risks of cardiovascular mortality (14). Thus, self-evaluation of sleep is also useful and noteworthy in public health management.

Some researchers evaluated sleep disorders by asking participants whether they had ever told a doctor they had trouble sleeping (15–17), in which the medical help seeking behavior reflected the severity of the sleep disorder. Therefore, in our study, self-reported sleep disturbance referred to the condition of ever consulting a doctor or other professionals because of trouble sleeping, which was reported to be prevalent in 19.2%–33.2% of US adults (18). It is no doubt that self-reported sleep disturbance seriously affects daily life, but whether the presence of such a sleep disorder affects long-term outcomes has not been investigated.

In the present study, we aimed to depict the prevalence of self-reported sleep disturbance and evaluate the association of self-reported sleep disturbance with all-cause and disease-specific mortality based on the National Health and Nutrition Examination Survey (NHANES) 2005 to 2018 dataset with linkage to the National Death Index (NDI) mortality files.

## Methods

### Study Population

The NHANES is a continuous program to assess the health and nutritional status of the civilian population in the United States.; it is conducted by the National Center for Health Statistics (NCHS), which belongs to the U.S. Centers for Disease Control and Prevention (CDC). Details about the NHANES study design, study protocol, and data collection have been described elsewhere (19). NHANES is approved by NCHS Ethics Review Board and written informed consent was obtained from all participants. The data in the present study were publicly released and were used in compliance with its data usage guidelines.

The present study population was limited to seven cycles of continuous NHANES (2005–2018). After excluding individuals less than 18 years old, during pregnancy, and without definite responses about sleep disorder, the remaining participants were included in the final analysis.

### Assessment of Self-Reported Sleep Disturbance

The questionnaire about sleep disorder (SLQ) has been added to NHANES since 2005, so we extracted sleep disorder data from 2005. In all the seven cycles of NHANES (2005–2018), the specific question, SLQ050, “Have you ever told a doctor or other health professionals that you have trouble sleeping?” was asked, and those participants responding “Yes” were considered to have self-reported sleep disturbance; participants responding “No” were considered not to have self-reported sleep disturbance; participants who chose “Refused” or “Don’t know” or did not answer this question were excluded in our analysis.

### Ascertainment of All-Cause Mortality and Disease-Specific Mortality

All-cause and disease-specific mortality were determined by linking to the NDI (20), in which the public-use Linked Mortality Files (LMF) are available for 1999–2018 NHANES. Death from cardiovascular disease (CVD) was defined as codes I00–I09, I11, I13, I20–I51, and I60–I69; death from cancer was defined as codes C00-C97; death from chronic lower respiratory disease (LRD) was defined as codes J40-J47, according to the 10th revision of the International Statistical Classification of Diseases, Injuries, and Causes of Death (ICD-10) guidelines.

The follow-up time has been calculated using months from the date of the interview to the date of death or the end of the mortality period (31 December 2019) for each participant of the NHANES.

### Assessment of Other Covariates

Demographics, basic physical examination (namely body mass index, BMI, calculated as weight in kilograms divided by the square of height in meters), behavioral factors, and chronic conditions collected when enrolled into the NHANES (baseline), along with an interview regarding sleep problems, were analyzed as covariates based on previous literature to reduce the effect of potential confounding (21–23).

Demographic covariates included age (years), sex (male, female), race (Mexican American, non-Hispanic white, non-Hispanic black, and others), education (less than 9th grade, 9–11th grade, high school, college or AA degree, college graduate or above), marital status (married, widowed, divorced, separated, never married, and living with partner), and family income to poverty ratio (PIR, a ratio of family income to poverty threshold, continuous from 0 to 4.99, values are 5 if the ratio is 5 or over).

Behavioral covariates included cigarette smoking (defined as never, former, and now smoker, based on responses to the following two questions: “Have you smoked at least 100 cigarettes in your entire life?” and “Do you now still smoke cigarettes?”); alcohol use, defined as never (had <12 drinks in lifetime), former (had ≥12 drinks in lifetime but did not drink last year), mild (≤1 drink per day for women or ≤2 drinks per day for men), moderate (≤2 drinks per day for women or ≤3 drinks per day for men), and heavy (>2 drinks per day for women or >3 drinks per day for men); caffeine consumption (evaluated as the mean consumption from 2 typical days and recorded in mg/day) (24); and overall diet quality (assessed by the Healthy Eating Index, HEI-2015) (25).

Chronic comorbidity conditions, namely hypertension, diabetes, and coronary heart disease (CHD) were obtained through questionnaires, based on the response of participants to the question of whether or not they were ever told by a doctor or other health professionals that they had a specific disease.

### Statistical Analyses

Statistical analyses were performed according to NHANES recommended guidelines (26). Since NHANES used a complex, multistage probability sampling design to select participants, and generated a representative sample of the US civilian non-institutionalized resident population, this survey design was considered and survey sample weights were used in most of our analyses unless otherwise noted. Data are presented as survey-weighted mean (95% confidence intervals, CIs) for continuous variables, or number (survey-weighted percentage, %) for categorical variables, respectively. Survey-weighted linear regression (svyglm) and survey-weighted Chi-square test (svytable) were used to detect statistical differences between the means and proportions between the two groups.

Univariate and multivariate Cox regression models were applied to evaluate the association between sleep disorders and mortality. For multivariate regression models, collinearity diagnostics were performed to ensure variance inflation factors (VIF) of all independent variables less than 5; then multiple stepwise regression was performed to determine the inclusion of each covariate. The crude and adjusted hazard ratios (HRs) and 95% CIs were calculated for the risk of sleep disturbance associated with all-cause or disease-specific death.

According to the recommendation of the Strengthening the Reporting of Observational Studies in Epidemiology (STROBE) statement (27), we simultaneously showed the results unadjusted and with different covariate adjustment strategies in three models. Minimally adjusted models (Model 1) were adjusted for age, sex, and race. Partially adjusted models (Model 2) were additionally adjusted for education, marital status, PIR, BMI, smoking, alcohol use, caffeine consumption, and HEI-2015. Fully adjusted models (Model 3) were additionally adjusted for common chronic comorbidities, namely hypertension, diabetes, and CHD.

The stratified analyses were performed to demonstrate the effect of self-reported sleep disturbance in different subgroups using the fully adjusted model except for the specific stratification variable and interactions of self-reported sleep disturbance with stratification variables, which were inspected by the likelihood ratio test.

Data were analyzed with the use of the statistical packages R (The R Foundation; http://www.r-project.org; version 4.2.0) and EmpowerStats (www.empowerstats.net, X&Y solutions, Inc. Boston, Massachusetts; version 4.1). A two-sided *p* < 0.05 was considered significantly different.

## Results

### Selection of Participants

**FIGURE 1:**
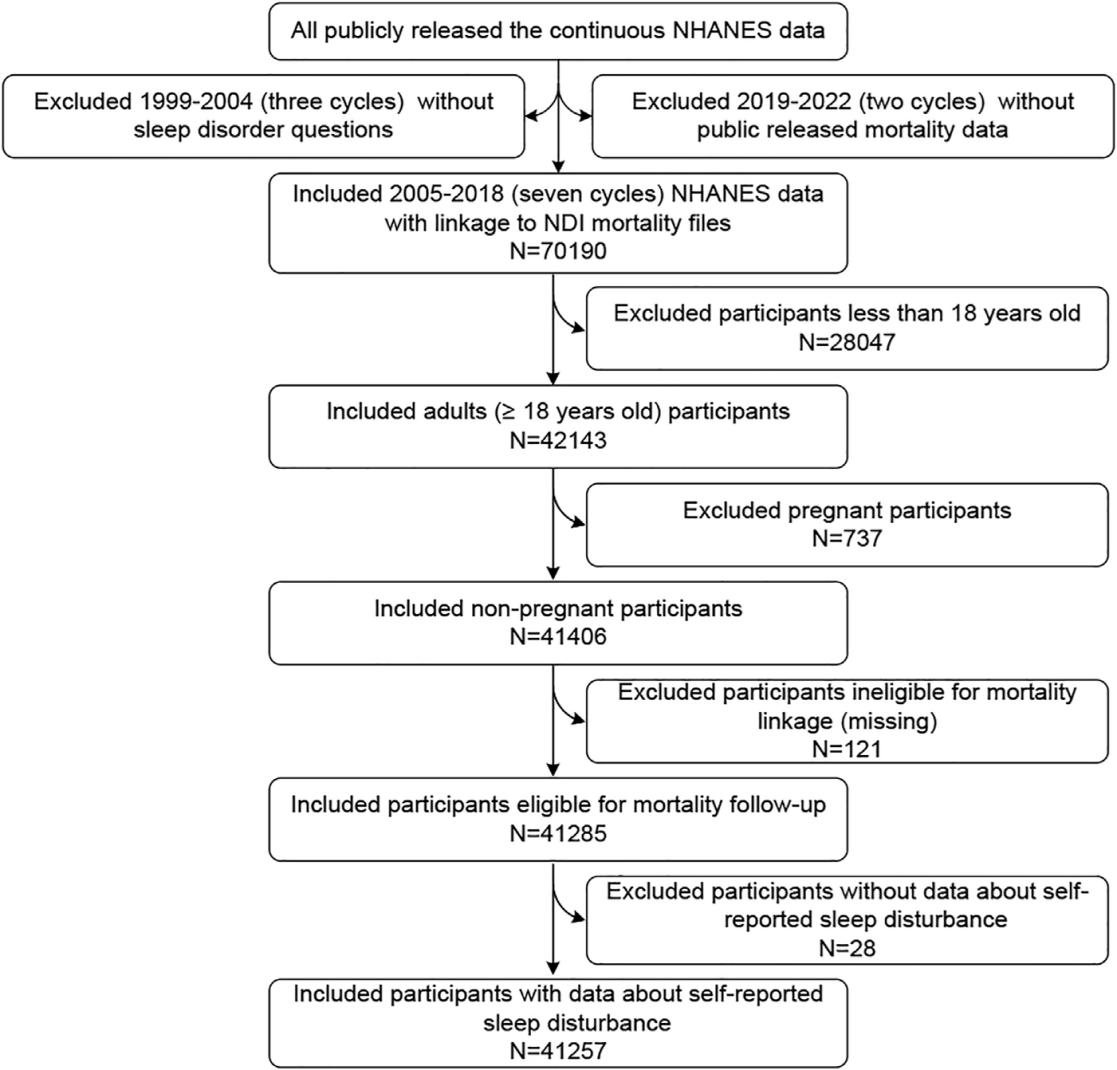
Flowchart of the present study indicating the included and excluded participants (National Health and Nutrition Examination Survey, the United States, 2005–2018).

The total number of participants in 7 cycles of NHANES (2005–2018) was 70,190. After excluding 28,047 individuals with ages less than 18 years old, another 737 pregnant individuals, another 121 individuals ineligible for mortality follow-up (insufficient identifying data), and another 28 responding to the question, “Have you ever told a doctor or other health professional that you have trouble sleeping?”, with “Refused” or “Don’t know” or no response, 41,257 participants were finally included in our study (Figure 1).

### Baseline Characteristics of Participants and Sleep Characteristics Based on the Presence of Self-Reported Sleep Disturbance

The enrolled 41,257 participants represented 228.6 million US adults. The survey-weighted mean age was 46.6 years old (95% CI: 46.2–47.1). The cohort comprised 20,897 women (survey-weighted percentage, 51.1%); 16,879 non-Hispanic White (66.6%), 9,063 non-Hispanic Black (11.4%), 6,595 Mexican American (8.6%), and 8,702 participants of other ethnicities (13.4%) (Table 1).

**TABLE 1:** Baseline characteristics of participants (National Health and Nutrition Examination Survey, the United States, 2005–2018).

Variables	Self-reported sleep disturbance			
	Total (n = 41,257)	No (*n* = 31,188)	Yes (*n* = 10,069)	P-value
Sex				<0.001
Female	20,862 (50.6)	14,981 (48.4)	5,881 (58.6)	
Male	20,395 (49.4)	16,207 (51.6)	4,188 (41.4)	
Age (years)	46.6 (46.2, 47.1)	45.2 (44.7, 45.7)	50.5 (49.9, 51.0)	<0.001
Race				<0.001
White	16,897 (41)	11,803 (63.7)	5,094 (74.4)	
Black	9,063 (22)	6,937 (11.9)	2,126 (10.1)	
Mexican	6,595 (16)	5,506 (9.9)	1,089 (5.1)	
Other	8,702 (21.1)	6,942 (14.5)	1760 (10.4)	
Education				<0.001
Less than 9th grade	4,309 (11.1)	3,401 (6.1)	908 (4.6)	
9–11th grade	5,570 (14.3)	4,225 (11.0)	1,345 (9.7)	
High school graduate/GED	8,932 (23)	6,634 (23.1)	2,298 (24.2)	
Some college or AA degree	6,776 (17.4)	4,790 (17.9)	1986 (21.5)	
College graduate or above	13,255 (34.1)	10,001 (41.9)	3,254 (40.0)	
Marital status				<0.001
Never married	7,583 (19.2)	5,984 (19.6)	1,599 (15.5)	
Living with partner	3,121 (7.9)	2,401 (8.2)	720 (7.7)	
Married	19,768 (50.1)	15,180 (55.8)	4,588 (51.9)	
Separated	1,325 (3.4)	909 (2.2)	416 (3.1)	
Divorced	4,266 (10.8)	2,781 (8.8)	1,485 (14.1)	
Widowed	3,361 (8.5)	2,320 (5.4)	1,041 (7.7)	
PIR	3.0 (2.9, 3.0)	3.0 (2.9, 3.0)	3.0 (2.9, 3.1)	0.951
BMI (kg/m^2^)	28.9 (28.7, 29.1)	28.4 (28.3, 28.6)	30.1 (29.9, 30.4)	<0.001
Smoker				<0.001
Never	22,263 (56)	17,611 (58.8)	4,652 (46.5)	
Former	9,381 (23.6)	6,586 (22.3)	2,795 (29.4)	
Now	8,126 (20.4)	5,651 (18.9)	2,475 (24.0)	
Alcohol user				<0.001
Never	5,183 (15.3)	4,177 (12.6)	1,006 (8.7)	
Former	5,478 (16.2)	3,778 (12.1)	1700 (16.2)	
Mild	11,173 (33)	8,272 (35.8)	2,901 (37.1)	
Moderate	5,147 (15.2)	3,767 (17.0)	1,380 (18.4)	
Heavy	6,861 (20.3)	5,285 (22.5)	1,576 (19.6)	
Caffeine consumption (mg/day)	167.3 (162.8, 171.7)	161.4 (156.4, 166.3)	182.8 (177.1, 188.6)	<0.001
HEI-2015	50.4 (50.1, 50.8)	50.6 (50.2, 51.0)	50.1 (49.7, 50.5)	0.03
Hypertension				<0.001
No	26,947 (65.4)	21,882 (73.6)	5,065 (55.9)	
Yes	14,248 (34.6)	9,257 (26.4)	4,991 (44.1)	
Diabetes				<0.001
No	35,196 (85.4)	27,317 (90.5)	7,879 (83.2)	
Borderline	899 (2.2)	584 (1.7)	315 (2.9)	
Yes	5,131 (12.4)	3,265 (7.7)	1866 (13.9)	
CHD				<0.001
No	37,099 (95.8)	27,994 (97.2)	9,105 (94.79)	
Yes	1,637 (4.2)	998 (2.8)	639 (5.21)	

Continuous variables are presented as survey-weighted mean (95% CI) and categorical variables are presented as number (survey-weighted percentage, %).

CI, confidence interval; BMI, body mass index; GED, general educational; AA, associate of arts; PIR, family income-to-poverty ratio; HEI, healthy eating index; CHD, chronic heart disease.

The self-reported sleep disturbance was estimated to affect 27.0% (95% CI: 26.1%–27.8%) of the whole population. Table 1 also shows the demographics, behavioral variables, and chronic comorbidities in all participants stratified by those with or without the self-reported sleep disturbance. Subjects reporting sleep disturbance were likely to be older (50.5 vs. 45.2), female (58.6% vs. 48.4%), white (74.4% vs. 63.7%), smoker (22.6% vs. 18.1% for current smoker and 28.1% vs. 21.5% for former smoker), with higher BMI (30.1 vs. 28.4), diabetes (13.0% vs. 7.3%), hypertension (42.5% vs. 25.4%), and CHD (5.2% vs. 2.8%) at the time of their participation in NHANES, than those not complaining about sleep.

To depict the detailed manifestations of self-reported sleep disturbance, general sleep symptoms were compared according to whether participants had self-reported sleep disturbance. As shown in Table 2, it was more common for individuals with self-reported sleep disturbance to have shorter sleep duration, to have trouble falling asleep, to wake up during the night, to wake up too early in the morning, to snore or stop breathing during sleep, to take pills to help sleep, to feel like they are not getting enough sleep, to feel unrested and overly sleepy during the day.

**TABLE 2:** The weighted sleep characteristics of participants with or without self-reported sleep disturbance (National Health and Nutrition Examination Survey, the United States, 2005–2018).

	Self-reported sleep disturbance		
	No, mean (95% CI) or n (%)	Yes, mean (95% CI) or n (%)	P-value
How much sleep do you get (hours)?	7.0 (7.0, 7.1)	6.5 (6.4, 6.6)	<0.001
How long to fall asleep (minutes)?	18.9 (18.2, 19.6)	29.8 (28.6, 31.0)	<0.001
How often have trouble falling asleep?			<0.001
Never	4,425 (45.9)	457 (16.9)	
Rarely (1 time a month)	1830 (23.7)	356 (16.7)	
Sometimes (2–4 times a month)	1720 (20.6)	653 (25.8)	
Often (5–15 times a month)	528 (6.5)	511 (20.5)	
Almost always (16–30 times a month)	334 (3.4)	526 (20.1)	
How often wake up during night?			<0.001
Never	4,020 (41.8)	440 (17.0)	
Rarely (1 time a month)	1809 (22.4)	312 (14.0)	
Sometimes (2–4 times a month)	1911 (22.8)	648 (26.0)	
Often (5–15 times a month)	737 (9.5)	600 (23.9)	
Almost always (16–30 times a month)	358 (3.6)	500 (19.2)	
How often wake up too early in morning?			<0.001
Never	4,546 (49.4)	640 (25.6)	
Rarely (1 time a month)	1,650 (20.5)	366 (17.0)	
Sometimes (2–4 times a month)	1,607 (18.6)	574 (24.0)	
Often (5–15 times a month)	669 (8.0)	517 (18.7)	
Almost always (16–30 times a month)	359 (3.5)	404 (14.6)	
How often do you snort or stop breathing?			<0.001
Never	13,224 (81.2)	3,372 (67.4)	
Rarely (1–2 nights/week)	1,577 (10.2)	697 (13.5)	
Occasionally (3–4 nights/week)	835 (5.0)	501 (8.9)	
Frequently—5 or more nights a week	588 (3.6)	542 (10.2)	
How often take pills to help you sleep?			<0.001
Never	7,952 (89.0)	1,413 (54.7)	
Rarely (1 time a month)	309 (4.0)	148 (6.4)	
Sometimes (2–4 times a month)	311 (3.8)	260 (11.4)	
Often (5–15 times a month)	101 (1.3)	205 (8.6)	
Almost always (16–30 times a month)	163 (1.9)	478 (18.8)	
How often did you not get enough sleep?			<0.001
Never	3,320 (30.3)	374 (12.5)	
Rarely (1 time a month)	1,634 (20.6)	325 (13.2)	
Sometimes (2–4 times a month)	2,258 (29.1)	682 (28.8)	
Often (5–15 times a month)	1,055 (13.6)	542 (23.6)	
Almost always (16–30 times a month)	547 (6.4)	570 (22.0)	
How often feel unrested during the day?			<0.001
Never	3,436 (32.0)	415 (13.2)	
Rarely (1 time a month)	1,486 (18.7)	285 (11.7)	
Sometimes (2–4 times a month)	2,313 (29.1)	666 (27.2)	
Often (5–15 times a month)	1,047 (14.0)	574 (25.2)	
Almost always (16–30 times a month)	541 (6.2)	562 (22.8)	
How often feel overly sleepy during day?			<0.001
Never	5,478 (26.0)	782 (11.6)	
Rarely (1 time a month)	4,081 (25.8)	929 (16.6)	
Sometimes (2–4 times a month)	4,965 (31.1)	1768 (32.2)	
Often (5–15 times a month)	1958 (12.7)	1,306 (25.2)	
Almost always (16–30 times a month)	851 (4.4)	847 (14.4)	
Have you ever been told by doctors to have sleep disorder?			<0.001
No	22,234 (97.9)	5,003 (73.8)	
Yes	432 (2.1)	1901 (26.2)	

Continuous variables are presented as survey-weighted mean (95% CI) and categorical variables are presented as number (survey-weighted percentage, %).

During a median follow-up of 88 months (interquartile: 49–129), 4,521 deaths were documented until 31 December 2019. Among them, 1,363 deaths were due to cardiovascular disease (CVD); 1,026 deaths were due to malignant neoplasms; 228 deaths were due to chronic lower respiratory diseases (LRD), and more details about the less common causes of death were shown in Supplementary Figure S1.

### Univariate Analysis of the Association of Self-Reported Sleep Disturbance With All-Cause and Disease-Specific Mortality

**FIGURE 2:**
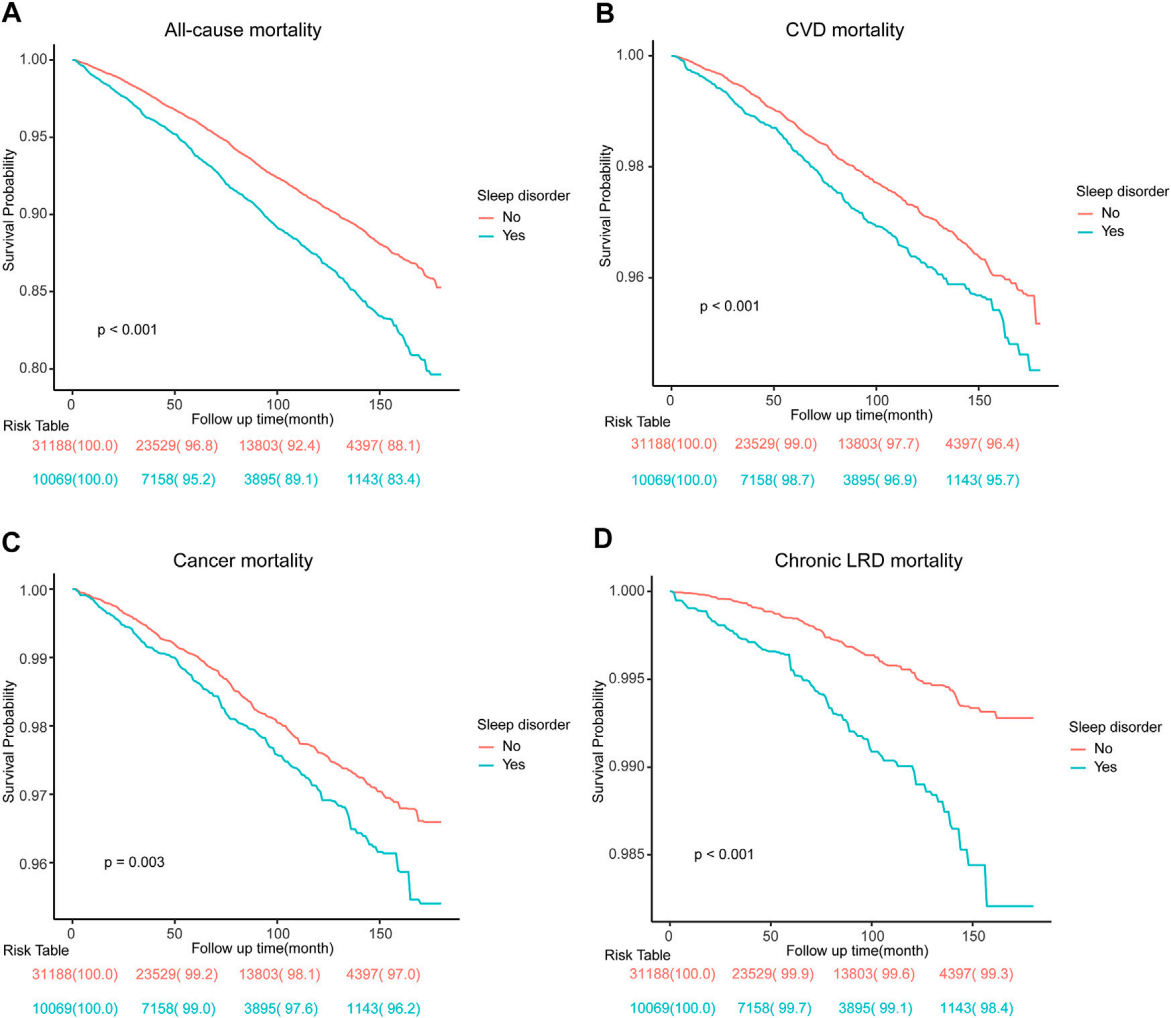
Survey-weighted Kaplan-Meier survival curves and log-rank tests comparing mortality due to all-cause **(A)**, cardiovascular disease **(B)**, cancer **(C)**, and chronic lower respiratory disease **(D)** in participants with or without self-reported sleep disturbance. CVD, cardiovascular disease; LRD, lower respiratory diseases (National Health and Nutrition Examination Survey, the United States, 2005–2018).

The unadjusted weighted Kaplan-Meier curves for all-cause mortality and disease-specific mortality by self-reported sleep disturbance were shown in Figure 2, followed by weighted log-rank tests. Then, univariate Cox regression models were used to quantify the risk of self-reported sleep disturbance with HR of 1.47 (95% CI: 1.36–1.59) for all-cause mortality, HR of 1.32 (1.13–1.54) for CVD mortality HR of 1.30 (1.10–1.53) for cancer mortality, and HR of 2.49 (1.85–3.35) for chronic LRD mortality (Supplementary Table S1). Univariate analysis of other covariates was also summarized in Supplementary Table S1.

### Multivariate Analysis of the Association Between Self-Reported Sleep Disturbance and All-Cause and Disease-Specific Mortality

After adjustment for age, sex, and race, self-reported sleep disturbance was associated with elevated risks of all-cause mortality (adjusted HR: 1.26, 95% CI:1.16–1.38), CVD mortality (1.17, 1.01–1.35), and chronic LRD mortality (2.08, 1.51–2.87), but no cancer mortality (1.13, 0.95–1.36) (Table 3).

**TABLE 3:** Multivariate analysis of the association between self-reported sleep disturbance and mortality (National Health and Nutrition Examination Survey, the United States, 2005–2018).

Outcome	Unadjusted		Model 1		Model 2		Model 3	
	HR (95% CI)	P-value	HR (95% CI)	P-value	HR (95% CI)	P-value	HR (95% CI)	P-value
All-cause mortality	1.47 (1.36, 1.59)	<0.001	1.26 (1.16, 1.38)	<0.001	1.23 (1.10, 1.38)	<0.001	1.17 (1.04, 1.32)	0.011
CVD mortality	1.32 (1.13, 1.54)	<0.001	1.17 (1.01, 1.35)	0.038	1.26 (1.03, 1.54)	0.023	1.19 (0.96, 1.46)	0.114
Cancer mortality	1.30 (1.10, 1.53)	0.002	1.13 (0.95, 1.36)	0.173	1.11 (0.91, 1.35)	0.312	1.10 (0.90, 1.35)	0.370
LRD mortality	2.49 (1.85, 3.35)	<0.001	2.08 (1.51, 2.87)	<0.001	2.04 (1.39, 3.00)	<0.001	1.88 (1.26, 2.80)	0.002

Data were calculated by svycoxph to fit a multivariate Cox proportional hazards model to data from a complex survey design.

Model 1: adjusted for sex, age, and race.

Model 2: adjusted for sex, age, race, education, marital status, family income-to-poverty ratio, BMI, smoker, alcohol user, caffeine consumption, HEI-2015.

Model 3: adjusted for sex, age, race, education, marital status, family income-to-poverty ratio, BMI, smoker, alcohol user, caffeine combustion; HEI-2015, comorbidity of hypertension, comorbidity of diabetes, comorbidity of coronary heart disease.

HR, hazard ratio; CVD, cardiovascular disease; LRD, lower respiratory diseases.

After further adjustment of other demographics and behavioral factors, namely education, marital status, PIR, BMI, smoking, alcohol use, caffeine consumption, and HEI-2015, self-reported sleep disturbance was associated with elevated risks of all-cause mortality (1.23, 1.10–1.38), CVD mortality (1.26, 1.03–1.54), chronic LRD mortality (2.04, 1.39–3.00), but no cancer mortality (1.11, 0.91–1.35, Table 3).

Finally, in the fully adjusted model of all covariates, with chronic comorbidities (diabetes, hypertension, CHD) included, self-reported sleep disturbance was still associated with elevated risks of all-cause mortality (1.17, 1.04–1.32) and chronic LRD mortality (1.88, 1.26–2.80), but not CVD mortality (1.19, 0.96–1.46) and cancer mortality (1.10, 0.90–1.35). As shown in Table 3, the associations in fully-adjusted models were largely attenuated, compared with that in non-adjusted models.

### The Stratified Analyses and Interaction Between Self-Reported Sleep Disturbance and Stratification Variables

**FIGURE 3:**
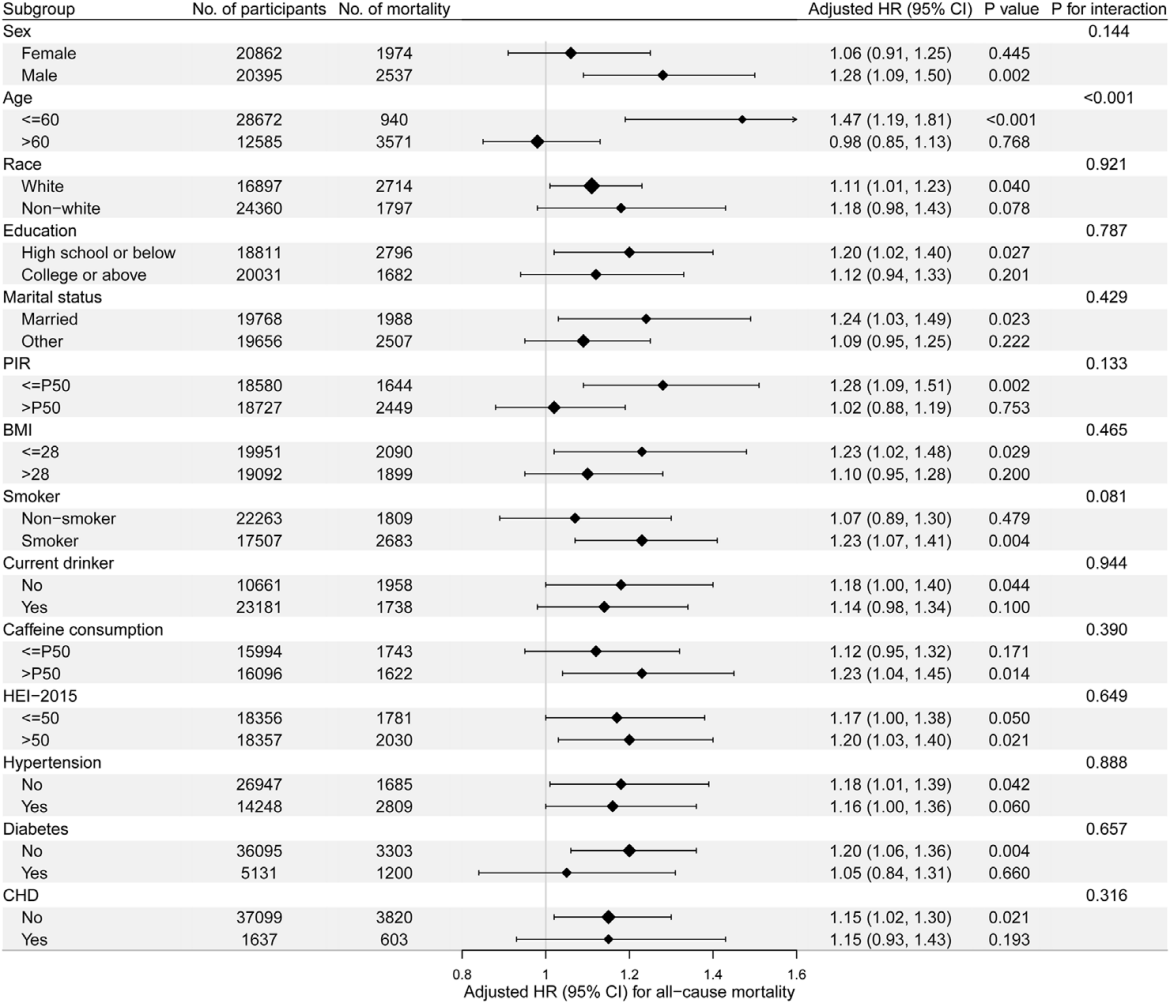
Stratified analyses of the associations of self-reported sleep disturbance and all-cause mortality and the interaction test between self-reported sleep disturbance and stratification variables. HR, hazard ratio; PIR, family income-to-poverty ratio; BMI, body mass index; HEI, Healthy Eating Index; CHD, coronary heart disease, P50, 50th percentile (National Health and Nutrition Examination Survey, the United States, 2005–2018).

Stratified analyses were performed to demonstrate the effect of self-reported sleep disturbance in different subgroups using the fully adjusted model, which was adjusted for sex, age, race, education, marital status, PIR, BMI, smoke, alcohol use, caffeine consumption, HEI-2015 and comorbidities of hypertension, diabetes, and CHD, except for the specific stratification variable of that analysis. The self-reported sleep disturbance was a significant risk factor for all-cause mortality in populations of males (adjusted HR: 1.28, 95% CI: 1.09–1.50), less than 60 years old (1.47, 1.19–1.81), married (1.24, 1.03–1.49), with an education level of high school or below (1.20, 1.01–1.23), with low PIR (1.28, 1.09–1.51), with low BMI (1.23, 1.02–1.48), smoker (1.23, 1.07–1.41), with more caffeine consumption (1.23, 1.04–1.45), and without diabetes (1.20, 1.06–1.36) (Figure 3).

The interaction analyses were performed to test the possible interaction of self-reported sleep disturbance with the stratification variable after adjusting all other variables. The interaction with the dichotomous age of 60 years old is remarkable (*p* < 0.001), indicating that self-reported sleep disturbance would be a more significant risk factor for all-cause mortality in the young adults than in the old population. The interactions with other variables are not statistically significant (Figure 3).

## Discussion

In this nationally population-based prospective cohort study, we demonstrated that self-reported sleep disturbance affected about 27% of US adults (approximately 61.7 million), more prevalent in populations who are older, female, white, and smokers, and those who have higher BMI, more caffeine consumption, diabetes, hypertension, and CHD. Individuals with self-reported sleep disturbance tended to have a higher risk of mortality from all-cause, CVD, cancer, and chronic LRD. After adjusting all covariates, the HR of self-reported sleep disturbance was largely attenuated, but it still was associated with a higher risk of mortality from all-cause and chronic LRD, suggesting the potential beneficial role to prevent and treat respiratory disease in individuals with self-reported sleep disturbance. There was potential interaction between self-reported sleep disturbance and age; the self-reported sleep disturbance tended to be associated with a higher risk of all-cause mortality in young adults than in old adults.

According to the International Classification of Sleep Disorders, third edition (ICSD-3), sleep disorders can be classified into insomnia, sleep-disordered breathing, central disorders of hypersomnolence, circadian rhythm sleep-wake disorders, parasomnias, and sleep-related movement disorders (28, 29). Insomnia is the most common type and affects nearly one-third of the adult population; and even if only taking into account insomnia with symptoms severe enough to cause daytime consequences, the prevalence is still more than 10% in adults and higher in women than men (17.6% vs. 10.1%) (30). The prevalence of other types of sleep disorders varied greatly based on the specific condition (30, 31). However, the conventional diagnosis of sleep disorder may underestimate the prevalence of sleep disorder, because of the delayed seeking of medical advice, complicated diagnostic procedures, and some uncategorized subtypes of sleep disorder (32, 33). If all sleep disorders that require consultation with doctors were taken as a whole, whether it could affect long-term outcomes and what the effect size would be, have not been explored.

In the present study, we demonstrated that self-reported sleep disturbance is prevalent in 27% of US adults, consistent with previous reports (18), and is associated with higher risks of mortality from all-cause by 47%, from CVD by 32%, from cancer by 30%, and from chronic LRD by 149%, regardless of other factors. To explore the independent role of self-reported sleep disturbance, we adjusted demographic, socioeconomic, lifestyle, and comorbidities conditions step by step, and we found the HRs of self-reported sleep disturbance were gradually attenuated. This was probably because sleep disturbance could be influenced by these behavioral factors and it could also be an accompanying symptom of some chronic comorbidities, such as diabetes (31, 34, 35), hypertension (17, 36–38), and CHD (39, 40). Actually, sleep intervention is already one of the recommended preventive and therapeutic strategies for such chronic diseases (41–44).

Many researchers have demonstrated that sleep disorders could promote the incidence of CVD and its mortality (7, 40, 45), possibly by increasing adverse cardiometabolic risk (39, 46), and healthy sleep behavior is recommended to promote ideal cardiac health, along with efforts to address other established risk factors including blood pressure, cholesterol, diet, blood glucose, physical activity, weight, and smoking cessation (46). However, in our fully adjusted model, the HR of self-reported sleep disturbance to CVD mortality did not reach the statistical significance level. This was probably because we have balanced major CVD mortality-related risk factors, such as BMI, hypertension, and CHD, which might be the main mechanism of sleep disorders affecting CVD mortality.

In the present study, we found that self-reported sleep disturbance was significantly associated with higher mortality from chronic LRD. It has been shown that the prevalence of sleep disturbance is higher in individuals with chronic respiratory disease, such as chronic obstructive pulmonary disease (COPD) (47) and asthma (48), which was probably because sleep disturbance was a premonitory or accompanying symptom of respiratory disease. Another possible explanation would be that sleep disturbances usually overlapped with some respiratory diseases (49–51), which was associated with a worse prognosis (52–54). Our results indicated that effective prevention and treatment of respiratory diseases may be worth advocating for in individuals with sleep disturbances.

In our stratified analysis and interaction analysis, we found that after all other covariates were adjusted, the association of self-reported sleep disturbance with all-cause mortality was more pronounced in a population of less than 60 years old and the interaction with age is statistically significant. This is consistent with previous research (45, 55), and the possible reasons are that bad habits, such as more sedentary time, more screen use time, and more sleep deprivation appear more in young adults and were not adjusted in the model; then similarly the higher prevalence of chronic diseases in senior adults weaken the effect of sleep disturbance since they were adjusted in the model. Recent research indicated that poor sleep behavior is independently associated with an increased risk of subclinical multi-territory atherosclerosis in middle-aged participants free of known CVD history (56), suggesting that sleep disturbance in the seemingly healthy population should be noticeable to public health practitioners.

Several possible mechanisms contributed to the mortality increase due to sleep disturbances, such as dysfunction of the autonomic nervous system (57, 58), endothelial function (59), metabolic regulation (60), inflammatory factors secretion (61–63), and arrhythmogenic effects on the heart (64, 65). However, due to the complexity and heterogeneity of sleep disturbance, more well-designed experimental research is needed to verify these mechanisms.

The strengths of our study included the nationally representative sampling of participants, the corresponding survey-weighted analyses, the relatively large sample size (*n* > 40,000), and the adjustment of many confounding factors, which made the findings applicable to the US adult population. In addition, this was the first study to treat self-reported sleep disturbance as an exposure factor and to evaluate its association with mortality as far as we know.

However, some limitations of the present study must be acknowledged. Firstly, the participants were sampled from US non-institutional civilians, so we should be cautious when attempting to apply the results to other populations. Secondly, the effect of sleep disturbance on health is chronic and continuous, so distributing those participants into the self-reported sleep disturbance group, whose sleep function was just disturbed by some transient factors, may lead to the underestimation of the correlation between sleep disturbance and mortality. Thirdly, the definition of self-reported sleep disturbance in the present study is dichotomous and somewhat simple, although facilitating its application in clinical management, and evaluation with a period of follow-up or objective measurement with more detailed classification is needed from the perspective of academic rigor. Finally, residual or unmeasured confounding cannot be completely ruled out despite every effort made to adjust the major confounding factors.

### Conclusion

Self-reported sleep disturbance is associated with a higher risk of all-cause mortality. Among the major leading causes of death, a higher risk of chronic LRD mortality was associated with the self-reported sleep disturbance. Future large-scale longitudinal studies with a more elaborate classification of sleep disturbances are still needed to clarify the cause-and-effect relationship. Finally, complaints about sleep disturbance should be paid attention to in public health evaluation; moreover, effective prevention and treatment of respiratory disease in individuals with sleep disturbances are recommended.

## Supplementary Material

Supplementary Figure S1 The distribution of leading causes of death in the enrolled participants with survey-weighted percentages and 95% confidence intervals (National Health and Nutrition Examination Survey, the United States, 2005–2018).

Supplementary Table S1 Univariate analysis of the association between covariates and all-cause and specific mortality (National Health and Nutrition Examination Survey, the United States, 2005-2018).
