# Supplementary material for: Self-Reported Sleep Disturbance is an Independent Predictor of All-Cause Mortality and Respiratory Disease Mortality in US Adults: A Population-Based Prospective Cohort Study
Source: Int J Public Health. 2023 Feb 14;68:1605538. doi: 10.3389/ijph.2023.1605538 (PMC9971003; doi:10.3389/ijph.2023.1605538)
Supplement: Supplementary file 1 [file Table1.DOCX]

**Table S1** Univariate analysis of the association between covariates and all-cause and specific mortality (National Health and Nutrition Examination Survey, the United States, 2005-2018)

| Characteristics | All-cause mortality | | CVD mortality | | Cancer mortality | | Chronic LRD mortality | |
| --- | --- | --- | --- | --- | --- | --- | --- | --- |
|  | HR (95%CI) | P-value | HR (95%CI) | P-value | HR (95%CI) | P-value | HR (95%CI) | P-value |
| **Self-reported sleep disturbance** |  |  |  |  |  |  |  |  |
| No | Reference | Reference | Reference | Reference | Reference | Reference | Reference | Reference |
| Yes | 1.47(1.36,1.59) | <0.001 | 1.32(1.13,1.54) | <0.001 | 1.30(1.10,1.53) | 0.002 | 2.49(1.85,3.35) | <0.001 |
| **Sex** |  |  |  |  |  |  |  |  |
| Female | Reference | Reference | Reference | Reference | Reference | Reference | Reference | Reference |
| Male | 1.12(1.03,1.22) | 0.01 | 1.13(0.97,1.32) | 0.1 | 1.36(1.16,1.60) | <0.001 | 0.86(0.58,1.28) | 0.46 |
| **Age, years** | 1.09(1.09,1.10) | <0.001 | 1.12(1.11,1.14) | <0.001 | 1.09(1.08,1.10) | <0.001 | 1.11(1.09,1.14) | <0.001 |
| **Race** |  |  |  |  |  |  |  |  |
| White | Reference | Reference | Reference | Reference | Reference | Reference | Reference | Reference |
| Black | 0.86(0.74,1.00) | 0.04 | 0.94(0.73,1.21) | 0.66 | 0.88(0.65,1.21) | 0.44 | 0.42(0.12, 1.41) | 0.16 |
| Mexican | 0.38(0.28,0.53) | <0.001 | 0.37(0.19,0.72) | 0.003 | 0.33(0.15,0.71) | 0.005 | 0.11(0.00,21.26) | 0.41 |
| Other | 0.47(0.38,0.58) | <0.001 | 0.47(0.30,0.73) | <0.001 | 0.47(0.28,0.78) | 0.003 | 0.37(0.15, 0.91) | 0.03 |
| **Education** |  |  |  |  |  |  |  |  |
| Less than 9th grade | Reference | Reference | Reference | Reference | Reference | Reference | Reference | Reference |
| 9-11th grade | 0.70(0.59,0.82) | <0.001 | 0.63(0.48,0.82) | <0.001 | 0.94(0.73,1.22) | 0.66 | 0.69(0.44,1.07) | 0.1 |
| High school graduate/GED or equivalent | 0.56(0.48,0.65) | <0.001 | 0.53(0.42,0.66) | <0.001 | 0.70(0.50,0.97) | 0.03 | 0.55(0.37,0.80) | 0.002 |
| Some college or AA degree | 0.45(0.38,0.53) | <0.001 | 0.37(0.28,0.50) | <0.001 | 0.63(0.46,0.87) | 0.01 | 0.33(0.19,0.57) | <0.001 |
| College graduate or above | 0.32(0.28,0.37) | <0.001 | 0.28(0.21,0.38) | <0.001 | 0.45(0.34,0.60) | <0.001 | 0.19(0.12,0.33) | <0.001 |
| **Marital status** |  |  |  |  |  |  |  |  |
| Never married | Reference | Reference | Reference | Reference | Reference | Reference | Reference | Reference |
| Living with partner | 1.09(0.78, 1.53) | 0.6 | 0.83(0.36, 1.90) | 0.66 | 1.31(0.66, 2.61) | 0.43 | 3.87(0.14,106.50) | 0.42 |
| Married | 1.92(1.56, 2.37) | <0.001 | 2.06(1.37, 3.11) | <0.001 | 2.30(1.42, 3.72) | <0.001 | 7.11(0.44,115.10) | 0.17 |
| Separated | 2.25(1.46, 3.46) | <0.001 | 3.43(1.83, 6.40) | <0.001 | 1.79(0.59, 5.40) | 0.3 | 12.00(0.66,218.90) | 0.09 |
| Divorced | 2.94(2.32, 3.72) | <0.001 | 2.35(1.42, 3.90) | <0.001 | 3.74(2.23, 6.25) | <0.001 | 14.56(0.98,216.79) | 0.05 |
| Widowed | 12.42(10.12,15.24) | <0.001 | 16.69(11.29,24.68) | <0.001 | 8.36(5.20,13.45) | <0.001 | 47.02(3.18,694.54) | 0.01 |
| **PIR** | 0.80(0.78,0.82) | <0.001 | 0.79(0.76,0.83) | <0.001 | 0.87(0.84,0.91) | <0.001 | 0.76(0.69,0.83) | <0.001 |
| **BMI** | 1.00(1.00,1.01) | 0.06 | 1.02(1.01,1.02) | <0.001 | 1.00(0.99,1.01) | 0.67 | 0.97(0.94,1.00) | 0.04 |
| **Smoker** |  |  |  |  |  |  |  |  |
| Never | Reference | Reference | Reference | Reference | Reference | Reference | Reference | Reference |
| Former | 2.26(2.07,2.48) | <0.001 | 1.85(1.61,2.12) | <0.001 | 2.77(2.26,3.40) | <0.001 | 8.23(4.49,15.09) | <0.001 |
| Now | 1.44(1.29,1.61) | <0.001 | 0.93(0.73,1.18) | 0.54 | 2.06(1.69,2.51) | <0.001 | 7.35(3.57,15.14) | <0.001 |
| **Alcohol user** |  |  |  |  |  |  |  |  |
| Never | Reference | Reference | Reference | Reference | Reference | Reference | Reference | Reference |
| Former | 1.65(1.47,1.86) | <0.001 | 1.33(1.12,1.58) | 0.001 | 2.49(1.75,3.54) | <0.001 | 3.42(1.78,6.56) | <0.001 |
| Mild | 0.64(0.56,0.74) | <0.001 | 0.59(0.49,0.70) | <0.001 | 1.04(0.72,1.50) | 0.85 | 0.79(0.38,1.65) | 0.53 |
| Moderate | 0.38(0.31,0.46) | <0.001 | 0.29(0.19,0.42) | <0.001 | 0.75(0.48,1.18) | 0.21 | 0.80(0.40,1.60) | 0.53 |
| Heavy | 0.35(0.29,0.42) | <0.001 | 0.20(0.12,0.32) | <0.001 | 0.53(0.34,0.82) | 0.004 | 0.35(0.11,1.18) | 0.09 |
| **Caffeine consumption (mg/day)** | 1.00(1.00,1.00) | 0.58 | 1.00(1.00,1.00) | 0.002 | 1.00(1.00,1.00) | <0.001 | 1.00(1.00,1.00) | <0.001 |
| **HEI-2015** | 1.01(1.00,1.01) | <0.001 | 1.01(1.01,1.02) | <0.001 | 1.01(1.00,1.02) | 0.003 | 1.00(0.99,1.01) | 0.56 |
| **Hypertension** |  |  |  |  |  |  |  |  |
| No | Reference | Reference | Reference | Reference | Reference | Reference | Reference | Reference |
| Yes | 3.85(3.62,4.10) | <0.001 | 4.98(4.38,5.66) | <0.001 | 3.29(2.80,3.87) | <0.001 | 3.84(3.00,4.93) | <0.001 |
| **Diabetes** |  |  |  |  |  |  |  |  |
| No | Reference | Reference | Reference | Reference | Reference | Reference | Reference | Reference |
| Borderline | 2.10(1.76,2.51) | <0.001 | 2.19(1.54,3.11) | <0.001 | 2.10(1.59,2.76) | <0.001 | 2.30(1.45,3.65) | <0.001 |
| Yes | 3.67(3.43,3.92) | <0.001 | 4.56(4.14,5.02) | <0.001 | 2.42(1.97,2.96) | <0.001 | 2.86(1.95,4.21) | <0.001 |
| **CHD** |  |  |  |  |  |  |  |  |
| No | Reference | Reference | Reference | Reference | Reference | Reference | Reference | Reference |
| Yes | 5.53(5.18,5.91) | <0.001 | 8.03(7.31,8.82) | <0.001 | 3.76(3.14,4.51) | <0.001 | 6.46(5.37,7.76) | <0.001 |

Data were calculated by svycoxph to fit a Cox's proportional hazards model to data from a complex survey design.

HR, hazard ratio; CVD, cardiovascular disease; LRD, lower respiratory diseases; PIR, family income-to-poverty ratio; BMI, body mass index; HEI, Healthy Eating Index; CHD, coronary heart disease.
