# Supplementary figures and images for: Self-Reported Sleep Disturbance is an Independent Predictor of All-Cause Mortality and Respiratory Disease Mortality in US Adults: A Population-Based Prospective Cohort Study
Source: Int J Public Health. 2023 Feb 14;68:1605538. doi: 10.3389/ijph.2023.1605538 (PMC9971003; doi:10.3389/ijph.2023.1605538)

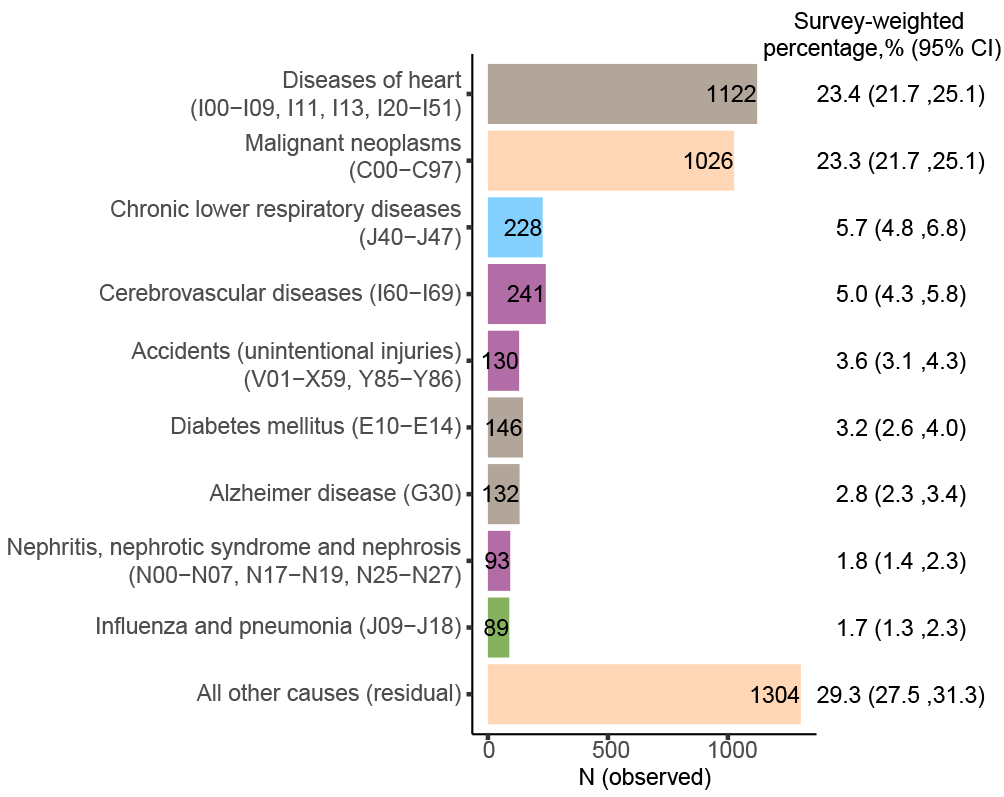

Supplement: Supplementary file 2 [file Image1.TIF]
